# Pharmacokinetics of Single Domain Antibodies and Conjugated Nanoparticles Using a Hybrid near Infrared Method

**DOI:** 10.3390/ijms22168695

**Published:** 2021-08-13

**Authors:** Shiran Su, Thomas J. Esparza, Duong Nguyen, Simone Mastrogiacomo, Joong H. Kim, David L. Brody

**Affiliations:** 1 Laboratory of Functional and Molecular Imaging, National Institute of Neurological Disorders and Stroke, National Institutes of Health, Bethesda, MD 20892, USA; shiran.su@nih.gov (S.S.); thomas.esparza@nih.gov (T.J.E.); dtn0028@gmail.com (D.N.); simone.mastrogiacomo@nih.gov (S.M.); caleb.kim@nih.gov (J.H.K.); 2 Department of Biomedical Engineering, Washington University in St. Louis, St. Louis, MI 63130, USA; 3 Center for Neuroscience and Regenerative Medicine, Henry M. Jackson Foundation for the Advancement of Military Medicine, Bethesda, MD 20892, USA; 4 Department of Neurology, Uniformed Services University of the Health Sciences, Bethesda, MD 20814, USA

**Keywords:** VHH, nanoparticles, near-infrared imaging, biodistribution, pharmacokinetic modeling

## Abstract

Iron oxide nanoparticles and single domain antibodies from camelids (VHHs) have been increasingly recognized for their potential uses for medical diagnosis and treatment. However, there have been relatively few detailed characterizations of their pharmacokinetics (PK). The aim of this study was to develop imaging methods and pharmacokinetic models to aid the future development of a novel family of brain MRI molecular contrast agents. An efficient near-infrared (NIR) imaging method was established to monitor VHH and VHH conjugated nanoparticle kinetics in mice using a hybrid approach: kinetics in blood were assessed by direct sampling, and kinetics in kidney, liver, and brain were assessed by serial in vivo NIR imaging. These studies were performed under “basal” circumstances in which the VHH constructs and VHH-conjugated nanoparticles do not substantially interact with targets nor cross the blood brain barrier. Using this approach, we constructed a five-compartment PK model that fits the data well for single VHHs, engineered VHH trimers, and iron oxide nanoparticles conjugated to VHH trimers. The establishment of the feasibility of these methods lays a foundation for future PK studies of candidate brain MRI molecular contrast agents.

## 1. Introduction

Neurological disorders affect millions of people worldwide, but at present our ability to assess these disorders objectively and quantitatively is limited [1]. Improvements in the assessment of neurological disorders would allow for disease progression monitoring and provide direct assessment of candidate therapeutics [2,3,4,5]. The long-term goal of this project is to develop MRI molecular contrast agents that will cross the blood brain barrier (BBB) and label relevant extracellular and intracellular biomarkers in the brain parenchyma. In the process of discovering and optimizing necessary components of these contrast agents, we have synthesized nanoparticles that consist of an iron oxide nanoparticle (IONP) core conjugated with Polyethylene glycol (PEG) plus single domain antibodies from camelids (VHH) for specific targeting.

IONPs have been widely used for medical applications including cancer diagnosis and treatment [6,7], treatment of iron deficiency anemia [6], enhanced blood pool and tumor MRI imaging [8,9], MRI molecular imaging [10,11,12,13], and magnetic resonance angiography (MRA) [14]. Sillerud et al. synthesized a novel MRI contrast agent by conjugating superparamagnetic oxide nanoparticles (SPIONs) with anti-amyloid-beta precursor protein (AβPP) antibodies to specifically target amyloid-beta plaques [15]. Iron oxide nanoparticles have also been functionalized with single-chain antibodies (scFv) against activated platelets for T_1_ and T_2_-weighted MRI of thrombi [10]. IONPs are considered relatively safe and do not induce cytotoxicity below 100 μg/mL in vitro [16]. MRI Molecular contrast agents based on iron oxide nanoparticles have good biocompatibility, at least in part because human blood and tissues are naturally rich in iron [17,18]. The FDA has approved an IONP, Ferumoxytol, for treatment of iron-deficiency anemia in patients with chronic kidney disease [19]. Ferumoxytol is also used off-label as a contrast agent for MR angiography in patients with impaired renal function as well [20] and no major safety concerns have been reported. In a widely cited publication, Kim et al. [9] demonstrated that homogenous size iron oxide nanoparticle cores for MR imaging could be synthesized at large scales. Their extremely small 3 nm iron oxide nanoparticles (ESIONs) were shown to have a high r_1_ relaxivity of 4.78 mM^−1^s^−1^ at 3T and low r_2_/r_1_ ratio of 6.12, which maximizes the T1 contrast effect. ESIONs were tested using in vivo MRI. After tail vein injection of ESION (2.5 mg Fe/kg), blood vessels were brightened on the T1-weighted MR images, confirming that ESIONs can enhance T1 relaxation and be used as a T1 MRI contrast agent. The iron oxide core of this prototype nanoparticle contrast agent was coated with PEG [21]. PEG is a common coating material that is used to prevent nanoparticle fouling in blood by reducing protein binding and to prolong circulation times by reducing clearance by the reticuloendothelial system (RES) [22].

Camelids, which include llamas, alpacas, and camels, produce functional antibodies devoid of light chains called heavy chain-only antibodies (HCAbs) [23,24]. The heavy chain of this kind of antibody is folded into three domains: the N-terminal domain that is variable in sequence, followed by a hinge region and two constant domains. HCAbs recognize their cognate antigen by one single domain, the VHH. VHHs have a very small size compared to other antibodies or functional antibody fragments. The molecular weight of a VHH is approximately 15 kDa, which is around 1/10 of a conventional IgG’s molecular weight, and about 50% of that of a single chain variable fragment (ScFv) [25,26]. VHHs have been used for in vivo imaging and therapeutics [25,26]. For example, Li et al. labeled anti-Aβ42 and anti-Tau VHHs with Alexa488 fluorescent dye and visualized extracellular Aβ and intracellular neurofibrillary tangles using 2-photon-microscopy [27]. Vandesquille et al. conjugated a VHH (R3VQ)-targeting Aß with gadolinium to allow MRI detection of Aβ in post-mortem mouse brain [28]. Rincon et al. used VHHs to lower Aβ levels with AAV-based delivery of anti-BACE1 VHH into the CNS of a cerebral amyloidosis mouse model [29]. VHHs against SARS-CoV-2, which could bind spike protein receptor binding domain, were recently developed as potential therapeutics for coronavirus outbreaks [30,31,32,33]. A humanized divalent VHH targeting von Willebrand factor (Caplacizumab) was recently approved by the FDA for treatment of acquired thrombotic thrombocytopenic purpura [34,35]. Importantly, VHHs show low immunogenicity risk profile before humanization [36]. For human therapeutic purposes, VHHs have been humanized to further lower the risk of immunogenicity [37]. For example, the safety of Caplacizumab has generally been good [38].

Pharmacokinetics (PK) is the study of drug absorption, distribution, metabolism, and excretion [39]. Pharmacokinetic and biodistribution characteristics are important parameters to consider when designing and testing novel nanoparticles to achieve an appropriate level of nanoparticles in the target tissue site. Nanoparticles with either extremely short or extremely long circulation time are generally considered non-optimal; nanoparticles with extremely short circulation time may not have enough time to penetrate target tissue sites, while nanoparticles with extremely long circulation time could cause off-target tissue toxicity and reduce signal-to-noise ratio due to background signal [40,41,42,43]. Thus, it is helpful to measure PK characteristics at an early phase in the project development so that this information can be used to optimize nanoparticle design.

Multiple approaches have been used to acquire PK and biodistribution data from in vivo experiments. Plasma and tissue sampling followed by inductively coupled plasma mass spectrometry (ICP-MS) are often used for in vivo distribution and PK studies. In prominent examples of this approach, Lankveld et al. assessed silver nanoparticles using ICP-MS to determine the silver content in organs for their tissue distribution study [44]. Xue et al. also used ICP-MS to study the tissue distribution of iron oxide nanoparticles in mice [45]. This approach, however, is invasive and requires sacrificing animals at multiple time points, which introduces inter-animal variations in data. Instead, various non-invasive imaging methods including PET, SPECT/CT, and MRI have been used to help trace distribution and clearance patterns [46,47,48]. While MRI may be an imaging modality of choice for diagnostic studies, it is expensive, requires lengthy anesthesia times for animals, and has relatively poor time resolution for use in PK studies. PET and SPECT are also relatively expensive imaging methods and raise radiation safety concerns. Near infrared (NIR) imaging has been proposed as an alternative method to study pharmacokinetics and tissue distribution to facilitate nanoparticle development [40]. Compared with the aforementioned methods, NIR is a less-expensive, faster, and safer method, which can be used to investigate nanoparticles’ in vivo behavior in appropriate small animal models [40]. PK models use a system of mathematical equations to describe drug pharmacokinetics. In this study, the PK model was used to describe the distribution and excretion of VHHs and nanoparticles in mice. We used a common type of PK modeling called compartment modeling, which is based on the simplifying assumption that each ‘compartment’ (e.g., kidney, liver) is homogenous. Compartment models have been widely used for oncology, disease diagnosis, and imaging studies [49,50]. This paper combined near infrared imaging and multi-compartment model to study the pharmacokinetics of VHHs and IONPs in vivo.

This study establishes a foundation to understand the PK of VHHs and IONPs. In this study, IONPs conjugated to VHHs and labeled with a NIR fluorescent dye were injected intravenously (IV) into mice. The PKs of VHHs alone and VHH-conjugated IONPs were monitored using a high resolution NIR scanner. Fluorescence signal change in mouse brain, kidney, and liver were imaged over a period of 2 days post injection to qualitatively and quantitatively understand the PK of our nanoparticles. A five-compartment PK model was constructed to describe the relationship between in vivo PK and hydrodynamic size following single bolus IV injection. It was found that increasing VHH and nanoparticle size results in a switch from kidney-dominant clearance to liver-dominant clearance, which is consistent with past findings. The use of mathematical PK models provides a quantitative method to study the relationship between PK parameters and nanoparticle/VHH sizes and can be used to explore the in vivo pharmacokinetics and distribution of other VHHs and nanoparticles for future studies. This PK model will be incorporated and modified for describing novel VHH-IONP in vivo PK characteristics during the ongoing development of future brain MRI molecular contrast agents.

## 2. Results

### 2.1. VHH and VHH-Conjugated Nanoparticle Characterization

VHH singlet and VHH triplet products had characteristics consistent with expectation. Twenty-two 1 mL elution fractions were collected from a Superdex 75 size exclusion chromatography column. Peak fractions of purified VHH singlet and VHH triplet were collected for FNIR conjugation. VHH singlet peaked at fraction 11 and VHH triplet peaked at fraction 9 (Figure S1a). Based on the protein size standards, VHH singlet and VHH triplet eluted from the columns as expected based on their calculated sizes of 12.7 kDa and 36.1 kDa, respectively.

Based on the dynamic light scattering (DLS) data, the IONP_PEG2000_ particles had a hydrodynamic radius of 6.4–6.7 nm before and 7.0–7.6 nm after conjugation with VHH triplet (Figure S1c). The 9th and 10th fractions from a Superose 6 Increase SEC column (optimized for larger particles) were used for IV injection and PK studies (Figure S1b). The IONP_PEG2000/750_ VHH triplet constructs had hydrodynamic sizes of 8.3–9.1 nm (batch 1) and 8.0–9.1 nm (batch 2) before, and 11.9–13.9 nm (batch 1) and 12.2–14.5 nm (batch 2) after conjugation with VHH triplet. The fifth, sixth, and seventh fractions from batch 1 and the sixth and seventh fractions from batch 2 from the Superose 6 Increase column were used for IV injection and PK studies (Figure S1b).

### 2.2. In Vivo Pharmacokinetic Study Using NIR

We optimized methods for NIR-based pharmacokinetic studies and then used these methods to acquire consistent in vivo pharmacokinetic data in mice. The NIR fluorescence images acquired using the Pearl system showed very low background fluorescence signal at 800 nm, confirming that in the NIR, the intrinsic autofluorescence at this wavelength of light in the animals was negligible. Autofluorescence at 700 nm was higher, so we focused on 800 nm channel measurements for these experiments (Figure S2). It has been reported that the use of different NIR dyes can affect biodistribution of NIR-labeled VHHs [51] and monoclonal antibodies [52]. We tested IR-680RD dye (IRDye^®^, Licor, Lincoln, NE, USA), IR-800CW dye (IRDye^®^, Licor, Lincoln, NE, USA), and FNIR dye-conjugated nanoparticles and confirmed that the dyes can affect the apparent biodistribution (Figure S3). Mice had lower background autofluorescence at 800 nm channel than the 700 nm channel, so IR-800CW dye and FNIR dye were preferred over IR-680RD dye (Figure S3a). Comparing the IR-800CW dye and the FNIR dye, the signal quality and consistency was higher for the FNIR dye (Figure S3b,c). The stability of FNIR dye was tested by conjugating FNIR dye with VHH singlet. FNIR dye presented very good NIR signal stability (Figure S4a) and the VHH singlet-FNIR conjugate was stable in size over 14 days (Figure S4b).

**Figure 1:**
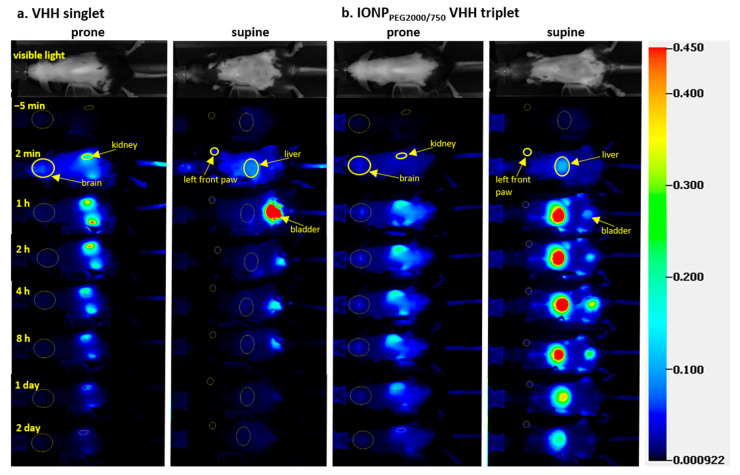
Representative in vivo serial fluorescence images and region of interest (ROI) analysis of singlet domain antibodies (VHH) singlet (**a**) and iron oxide nanoparticle_PEG2000/750_ (IONP_PEG2000/750_) VHH triplet (**b**). ROIs were drawn to track the fluorescence signal change of major ROIs including kidney and brain (prone position), left front paw, and liver (supine image) over time. Scale bar represents near-infrared (NIR) image intensity measured by Pearl. (**a**) In vivo serial fluorescence prone and supine position images before and 2 min, 1 h, 2 h, 4 h, 8 h, 1 day, and 2 days after IV bolus injection of VHH singlet. (**b**) In vivo serial fluorescence prone and supine position images before and 2 min, 1 h, 2 h, 4 h, 8 h, 1 day, and 2 days after IV bolus injection of IONP_PEG2000/750_ VHH triplet.

NIR images of in vivo mice injected with four different FNIR dye conjugates (two VHHs and two IONPs) showed that the conjugates had different biodistributions in brain, kidney, and liver and were cleared from mice at different rates (Figure 1). 

**Figure 2:**
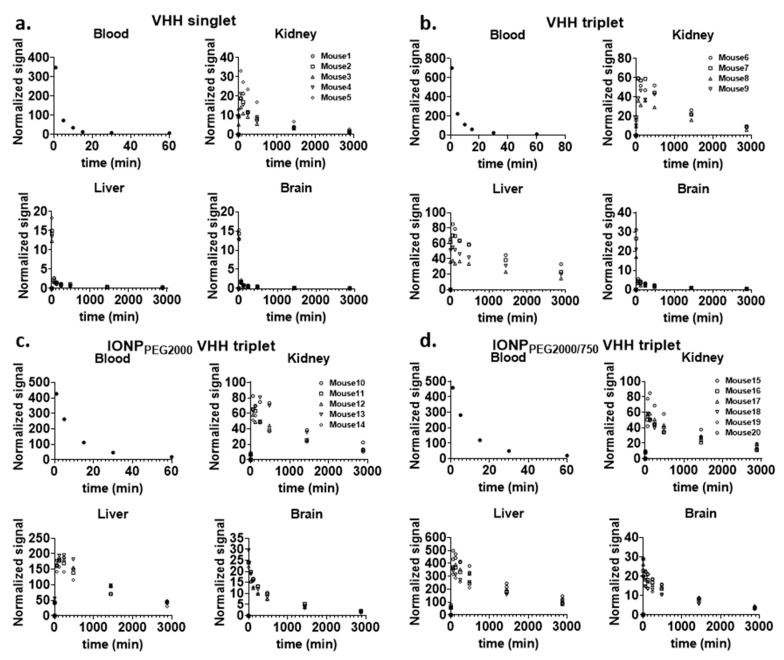
Major ROIs in vivo fluorescence signal changes over time. NIR signal change over time of major ROIs including blood, kidney, liver, and brain. (**a**) VHH singlet (five mice), (**b**) VHH triplet (four mice), (**c**) IONP_PEG2000_ VHH triplet (five mice), and (**d**) IONP_PEG2000/750_ VHH triplet (six mice) were injected by IV bolus, and the NIR signal changes were monitored in major ROIs.

Regions of interest (ROIs) of major organs including brain, kidney, and liver were drawn on the images acquired before, 2 min, 1 h, 2 h, 4 h, 8 h, 1 day, and 2 days after IV injection at both prone and supine positions of mice (Figure 1a for VHH singlet, Figure 1b for IONP_PEG2000/750_ VHH triplet). The reliability of ROI drawing method was calculated on five representative mice injected with VHH singlet (Figure S5). ROIs were drawn on the images two times on 2 different days. The Pearson correlation of the results from the two ROI drawings was very close to 1, indicating almost perfect reliability of the ROI drawing method. The fluorescence intensities of each organ were measured for PK analysis. Kidney intensities were multiplied by two to represent both kidneys. To analyze the relative NIR signal changes across mice injected with different FNIR dye conjugates, which had different absolute NIR intensities, the main ROI fluorescence signals were normalized using left front paw signals at 2 min post injection as internal controls values. We found this approach to be more reliable than using intrinsic fluorescence of the injected material or local tail fluorescence values. For VHH singlet, the pharmacokinetic plots of kidney and liver showed that kidneys and liver had similar uptake, but kidneys had much slower fluorescence intensity decrease rate than liver, indicating that kidneys were the major organ for VHH singlet clearance (Figure 2a). For VHH triplet, the pharmacokinetic plots of kidney and liver showed that kidneys and liver had similar uptake and clearance rates, indicating that kidneys and liver both contribute to the clearance (Figure 2b). For IONP_PEG2000_ VHH triplet and IONP_PEG2000/750_ VHH triplet, the pharmacokinetic plots of kidney and liver showed that liver had much larger uptake of nanoparticles than kidneys, indicating that liver was the main organ for the IONP-VHH conjugate clearance (Figure 2c,d). There were only modest differences in kinetics between the two different PEG coatings that were used to make the IONPs water soluble and serve as linkers for VHH conjugations. Particles coated with PEG2000 vs. particles coated with a 1:1 ratio of PEG2000 to PEG750 had similar kinetics with predominant liver uptake and biphasic clearance from the brain compartment.

**Figure 3:**
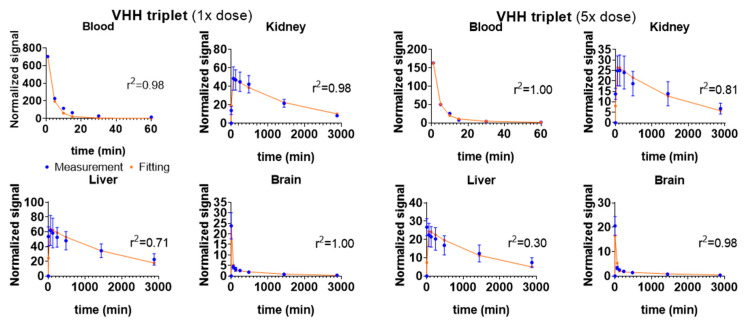
Dose-linearity testing: Fitting (red lines) of the five-compartment model solutions to the VHH triplet (1x dose) and VHH triplet (5x dose) measured data (blue symbols). The fitting-based kidney ratio and liver ratio are 37.18 and 33.11 for VHH triplet (5x dose) (*n* = 6), and 25.74 and 40.80 for VHH triplet (1x dose) (*n* = 4). Kidney ratio and liver ratio for VHH triplet (5x dose) and VHH triplet (1x dose) are of the same order and similar to each other. Error bars represent standard deviations.

The relationship between dose and kinetics was also explored using VHH triplet as an example (Figure 3). In this experiment, a higher dose VHH triplet at a concentration of 24.23 µM was intravenously injected into six mice and NIR images were taken at the same time points as in other experiments. Lower dose (4.75 µM) and higher dose (24.23 µM, ~5x lower dose) VHH triplet showed similar signal kinetics over all compartments. Higher dose and lower dose VHH triplet had very similar calculated kidney ratios and liver ratios, indicating dose-linearity of kinetics for VHH triplet.

All mice injected with VHH singlet, VHH triplet, IONP_PEG2000_VHH triplet, and IONP_PEG2000/750_ VHH triplet appeared healthy. No overt behavioral abnormalities were noted and veterinary intervention was not required.

### 2.3. Blood Clearance Measured by NIR

**Figure 4:**
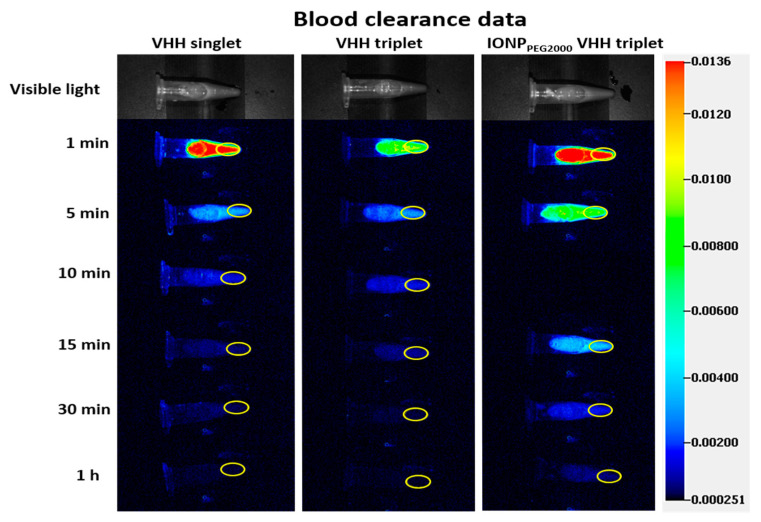
Ex vivo fluorescence images of blood. Scale bar represents NIR image intensity measured by Pearl. Mouse blood was collected at 1 min, 5 min, 10 min, 15 min, 30 min, and 1 h after intravenous (IV) injection of VHH singlet and VHH triplet. Mouse blood was collected at 1 min, 5 min, 15 min, 30 min, and 1 h after IV injection of IONP_PEG2000_ VHH triplet. ROIs were drawn for NIR signal analysis.

A limitation of the NIR fluorescence imaging approach was that we were unable to consistently assess the PK in blood. We initially tested the assumption that PK in peripheral tissues such as paw and tail would reflect PK in blood, but this turned out not to be correct; clearance from blood was substantially faster than peripheral tissues such as paw. Signal in ROIs containing heart and tongue muscle were too low to use for consistent model fitting [47,48] (Figure 1). Therefore, we adopted a hybrid approach and measured PK in blood by direct sampling of blood from separate groups of mice sacrificed at multiple time points. Blood was sampled at 1 min, 5 min, 10 min, 15 min, 30 min, and 1 h after IV injection and quantified using ROIs drawn on the NIR images of the ex vivo blood (Figure 4). 

**Figure 5:**
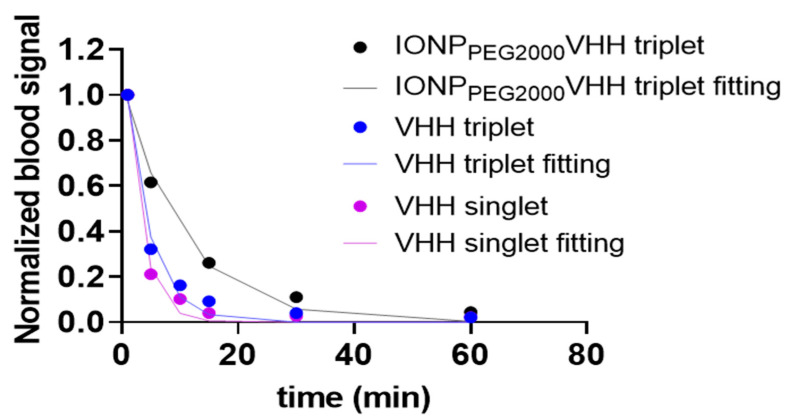
Blood clearance data and single exponential fitting. The NIR signals of VHH singlet, VHH triplet, and IONP_PEG2000_ VHH triplet in mouse blood after IV bolus injection. The NIR signals were measured at 1 min, 5 min, 10 min, 15 min, 30 min, and 1 h post injection for VHH singlet and VHH triplet. The NIR signals were measured at 1 min, 5 min, 15 min, 30 min, and 1 h post injection for IONP_PEG2000_ VHH triplet. The NIR blood signals were normalized to signals at 1 min post IV injection and fitted using the single exponential equation $y=a*e^{-\frac{x}{\tau }}$. The time constant τ best fit values were 2.80 min, 4.13 min, and 10.24 min for VHH singlet, VHH triplet, and IONP_PEG2000_ VHH triplet.

Then, the blood clearance curves were fitted using a single exponential equation (Figure 5). The NIR fluorescence images of ex vivo blood indicated fast blood clearance rate of VHHs and slower rate of VHH-IONP. Most of the materials were cleared out of the blood within the first 1 h post injection. The fitted half-lives were 7.09 min, 2.86 min, and 1.94 min for IONP_PEG2000_ VHH triplet, VHH triplet, and VHH singlet respectively.

### 2.4. Mathematical Modeling

**Figure 6:**
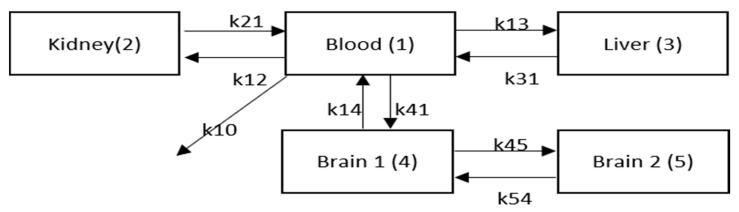
Schematic graph for the five-compartment mathematical model. This schematic graph describes the in vivo kinetics of VHHs/IONPs, including uptake, clearance and intercompartment exchanges. This model is characterized by a system of five ordinary differential equations (ODEs). k12, k21, k13, k31, k14, and k41 are the forward and reverse first-order transfer rate constants for the intercompartment change between blood compartment and kidney, liver, and brain compartments. k45 and k54 are the forward and reverse first-order transfer rate constants for intercompartment change between brain 1 and brain 2 compartments. k10 is the first-order rate constant for clearance from blood.

This mathematical PK model described the in vivo PK of FNIR-VHHs and FNIR-IONP-VHHs by segmenting the body into five main compartments and the five compartments were related through mass transfer (Figure 6). X1, X2, X3, X4, and X5 were the amounts of conjugates in blood, kidney, liver, brain 1, and brain 2 compartments. k12, k21, k13, k31, k14, and k41 were the forward and reverse first-order transfer rate constants for the intercompartment change between blood compartment and kidney, liver, and brain compartments. k45 and k54 were the forward and reverse first-order transfer rate constants for intercompartment change between brain 1 and brain 2 compartments. K10 was the first-order rate constant for clearance. The initial condition of blood compartment was used to describe the bolus IV injection to the blood compartment and set to be the normalized conjugate fluorescence intensity (normalized by left front paw signal intensity). The initial conditions of kidney, liver, paw, and brain compartments were set to be 0, based on the assumption that the nanoparticles/VHHs enter these organs only through the blood. The five ordinary differential equations (ODEs) were established to describe the mass transfer of VHHs/IONPs between the five compartments and their clearance.

(1) $$\frac{dX1}{dt}=-\left(k_{12}+k_{13}+k_{14}+k_{10}\right)X_{1}+k_{21}X_{2}+k_{31}X_{3}+k_{41}X_{4}$$

(2) $$\frac{dX2}{dt}=-k_{21}X_{2}+k_{12}X_{1}$$

(3) $$\frac{dX3}{dt}=-k_{31}X_{3}+k_{13}X_{1}$$

(4) $$\frac{dX4}{dt}=-(k_{41}+k_{45})X_{4}+k_{14}X_{1}+k_{54}X_{5}$$

(5) $$\frac{dX5}{dt}=-k_{54}X_{5}+k_{45}X_{4}$$

**Figure 7:**
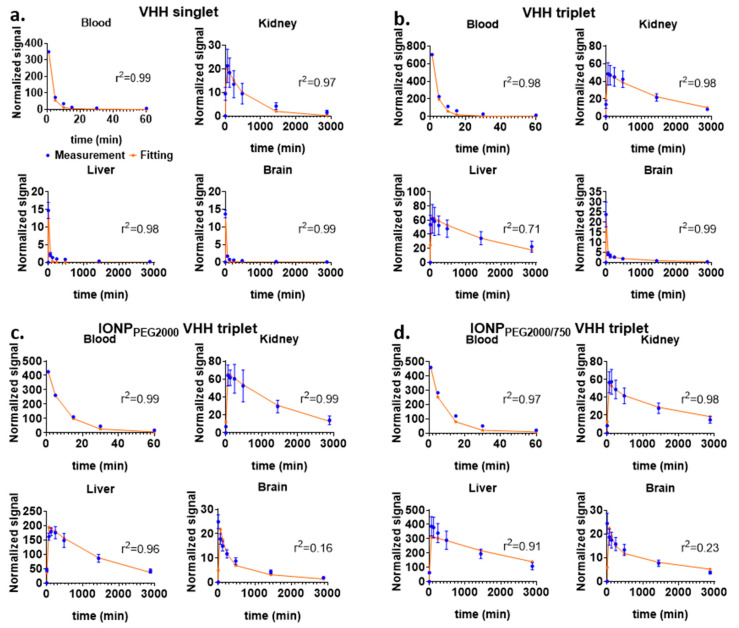
Fitting of the five-compartment model solutions to the experimentally measured fluorescence data. Fitting of the model simulated solutions (red line) and the measured normalized NIR signals (blue dot) of major organs after IV injection of VHH singlet (**a**), VHH triplet (**b**), IONP_PEG2000_ VHH triplet (**c**), and IONP_PEG2000/750_ VHH triplet (**d**). Error bars represent standard deviations.

The normalized averaged PK data were fitted using the five-compartment model. Figure 7 and Table 1 show the optimal model fitting for the two VHH constructs and the two VHH conjugated IONPs. The fitting corresponded well with the normalized data. A four-compartment model with only one brain compartment was tested first (Figure S6). However, with only one brain compartment, the fitting did not match the trend of brain signal change as well (Figures S7, and S8). A second brain compartment was added to better fit the brain signal change (Figure 6 and Figure 7). Small sample-corrected Akaike Information criteria (AICc) values were −35.90, −16.85, −20.47, and −41.29 for four compartment fitting and −41.59, −22.62, −27.48, −39.09 for five-compartment model fitting of VHH singlet, VHH triplet, IONP_PEG2000_ VHH triplet, and IONP_PEG2000/750_ VHH triplet respectively. Because models with lower AIC values are preferred, the five-compartment model was selected. No additional kidney or liver compartments were added because the benefit in terms of fitting was limited, and not justified based on the increased complexity of the model. Similarly, there was no additional benefits of adding kinetic parameters representing direct clearance from kidney (k20) or liver (k30) in terms of model fitting, and these rate constants could not be independently constrained by the acquired data. Therefore, these kinetic parameters were not included in the final models. For VHHs/nanoparticles that do not appreciably cross the BBB, PK of paw and brain compartments were similar (Figure S9), so the paw compartment was not included in the model separately. Table 1 shows the optimal sets of fitting parameters for the two VHHs and the two IONPs. Table 2 shows the r^2^ values and calculated kidney and liver uptake/clearance ratios, which were calculated by Equations (6) and (7).

(6) $$Kidney\;ratio=\frac{k_{12}}{k_{21}}$$

(7) $$Liver\;ratio=\frac{k_{13}}{k_{31}}$$

Comparing the values of kidney ratio and liver ratio, the kidney intake/clearance ratio was much larger than liver ratio for VHH singlet, the kidney and liver ratios were similar for VHH triplet, and the liver ratio was much larger than kidney ratio for both VHH-conjugated nanoparticles (Table 2). As expected, as the size increased from VHH singlet to IONP_PEG2000/750_ VHH triplet, the fitted liver ratio increased compared with the kidney ratio, replacing the kidney ratio’s dominant position. This is consistent with previous findings that when molecular size is below the renal filtration, molecules are mostly filtered out of the body through the kidneys. As molecular size increases and passes the renal filtration cutoff, liver plays a more substantial role in clearance.

### 2.5. Multidose Experiment and Model Fitting

**Figure 8:**
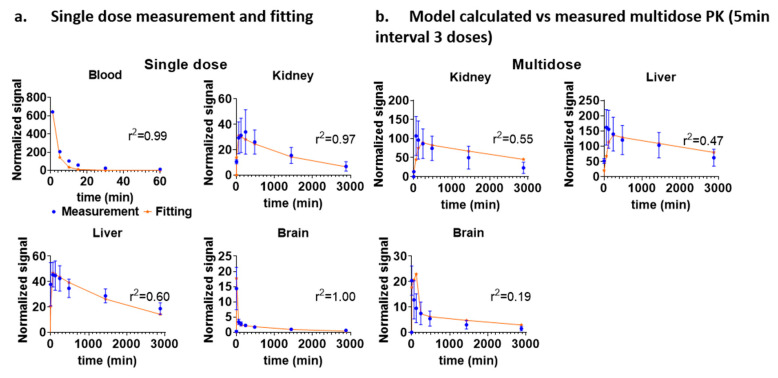
Prediction of multidose regimen using five-compartment model fitted using parameters from single-dose experiment. (**a**) NIR signal change over time after single IV bolus injection (*n* = 3 mice, blue dot) and the fitting solution (red line) calculated based on the five-compartment model. (**b**) Experimentally measured fluorescence signal (*n* = 5 mice, blue dot) and prediction of multidose signal (red line) based on the five-compartment model single dose fit with no free parameters. Three bolus IV injections with time intervals of 5 min between doses was performed for the multidose experiment. Error bars represent standard deviations.

As a test of the accuracy of the model, we used single dose-based kinetic parameters to predict the kinetics after multiple doses. An experiment with both single-dose and multidose IV injection was performed using VHH triplet. Single dose PK measurements were performed by intravenously injecting 0.1 mL VHH triplet into three mice and an additional group of five mice received three doses of the same 0.1 mL VHH triplet with time intervals of 5 min between doses. The normalized averaged single dose mice data was fitted using the five-compartment model. The five-compartment model fitted the single dose data well, with r^2^ values of 0.9947, 0.9747, 0.5998, and 0.9995 for blood, kidney, liver, and brain compartment, respectively. Then the predicted multidose kinetics were calculated based on the model with no additional free parameters through superposition. Finally, the single dose model-based predicted multidose kinetics were compared with the measured multidose kinetics (Figure 8a). The model-based predicted multidose kinetics closely followed the trend of the experimentally measured multidose kinetics (Figure 8b). Thus, the multidose data was moderately well fit by the model based on PK parameters derived from the single dose data, with r^2^ values of 0.5529, 0.4701, and 0.1855 for the kidney, liver, and brain compartments.

## 3. Discussion

In summary, we found that the kinetics of VHHs and VHH-conjugated iron oxide nanoparticles were clearly related to their size. The smallest sized VHH singlets were cleared mostly by the kidneys. As the size increased, the liver became progressively more dominant in the uptake and clearance of VHHs/nanoparticles. These findings are consistent with the hypothesis that the NIR imaging for PK study can provide information about relative nanoparticle/VHH concentration changes in mouse tissues and are in line with previous knowledge about renal filtration. It is known that the threshold of glomerular filtration for macromolecules and nanoparticles is between 5 and 8 nm with progressively decreasing filtration as molecular size increases [53,54,55]. The PK model constructed in this study had five compartments: blood, kidney, liver, brain, and brain extracellular. For model fitting, blood compartment kinetic data were needed. However, NIR signal from the blood compartment could not be directly measured using the Pearl system in vivo. Ex vivo experiments were therefore performed to understand the blood NIR signal change after IV injection of VHHs and to help with the model fitting. Thus, this approach should be considered a hybrid, with direct sampling of blood, and serial NIR imaging-based sampling of other compartments.

This study found that the relative value of uptake/clearance of liver and kidney ratios based on the fitted PK parameters can be used as reference to understand the relationship between VHH/nanoparticle sizes and in vivo behavior. Because we are most interested in nanoparticle/VHH’s kinetics in the brain for future nanoparticle/VHH brain targeting experiments, a second brain compartment was added to the model. This model describes the nanoparticle/VHH kinetics in the brain more accurately than a model with a single brain compartment. We demonstrated the robustness of the fitted PK model by testing linearity (Figure 3) and multidose conditions (Figure 8).

The VHHs and nanoparticles characterized in this study are prototypes for the design of the final contrast agents. Because we have not yet achieved BBB crossing, the binding of the VHHs and nanoparticles to their targets in the brain is not described in this paper. Instead, to facilitate development of the final contrast agents with optimal delivery capacity and biosafety, this study focused on understanding the relationship between in vivo pharmacokinetic (PK) characteristics and size of these prototype VHHs and nanoparticles.

It was found that in the NIR range of around 650 nm to 900 nm wavelength, biological tissues have the lowest absorption coefficient and minimum tissue autofluorescence [56,57]. Fluorescence dyes with excitation/emission wavelength in the near-infrared range have deeper penetration depth than visible light and provide higher signal to background ratio with decreased background noise and modest autofluorescence [58,59,60]. The advantage of our study is that it uses NIR to capture images from individual mice post IV injection over time. The time-series images collected from individual mice reduce variations between mice and improve data consistency. Also, the number of mice used was greatly reduced compared with the conventional methods for PK data collection (e.g., ICP-MS measurement of iron content). For a conventional ICP-MS-based PK study, multiple animals need to be sacrificed to collect data at each time point [44,45]. In our NIR imaging-based PK experiment, 20 mice were used to collect the main data: five mice for each of four VHH or VHH-nanoparticle constructs. To get the same amount of data, a conventional PK study would have required 140 mice (five mice × four constructs × seven time points) in total. NIR imaging also has the advantages of fast imaging speed, low cost, and modest regulatory oversight requirements compared to other imaging methods used for PK studies such as PET, SPECT, and MRI. A limitation of NIR imaging approaches in the past has been the quality of dyes available. Compared with commercially available dye IR-800CW, the FNIR we used has advantages including reduced aggregation and dramatically increased NIR emission brightness [61]. FNIR dyes have been used to label monoclonal antibodies to visualize the biodistribution and clearance following IV injection in mice [52].

Modeling pharmacokinetics with mathematical models helps with decision making in nanoparticle development. Pharmacokinetic modeling has been widely used to guide drug and nanoparticle development [62,63]. Compartment models are designed to simplify the complex processes related to drug distribution and elimination in the body [64]. In compartment models, drug tissue concentration is assumed to be uniform within a given hypothetical compartment. Tissues with similar PK characteristics are lumped into one hypothetical compartment. Compartment models have been used to understand drugs and nanoparticles in vivo PK [65,66,67]. Gadkar, K., et al. constructed a two-compartment model to guide antibody selection for Aβ reduction [62]. Uno, Y., et al. constructed a three-compartment model to estimate the interstitial concentration of talaporfin sodium [66]. Sim, H., et al. established a two-compartment model to study the relationship between tumor growth and drug uptake kinetics [68]. In this study, we established a multi-compartment model based on NIR images, which describes the PK of VHHs and nanoparticles in blood as well as other tissue compartments. This multi-compartment model will be used to guide future nanoparticle development and the compartments can be adjusted based on the study focus.

One limitation of this study is that the signal from NIR images does not reflect the absolute concentrations in the compartments of interest. The presence of skin and soft tissues reduces image quality by optical attenuation and scattering [59,60]. We propose that fluorescence signals provide information about the relative concentration kinetics in major ROIs, rather than exact concentration values. In addition, NIR methods are less amenable to assessing smaller compartments such as spleen or bone marrow. Clearly, NIR approaches are best suited for relatively short-term studies in small animals such as mice with little intrinsic skin pigmentation; in larger animals there is too much attenuation between the tissues of interest and the detectors. Longer term PK studies would be difficult because of the challenges of maintaining hair removal for more than a few days without compromising health.

Another substantial limitation is that we only characterized PK in relation to construct size. There are many other properties of IONPs including hydrophobicity, surface charge, and coating or conformation of nanoparticles/VHHs that could affect their PK and biodistribution [6,8,22,45]. We found minimal differences between IONPs coated with PEG2000 vs. a 1:1 ratio of PEG2000 to PEG750. Thus, there does not seem to be a major effect of the length of PEG coating in this case. Furthermore, the nanoparticles tested in this study are not our final product, and the binding properties of next generation nanoparticles will certainly influence the in vivo PK and will require modification of the model.

We acknowledge that we have not performed blood measurements for both IONP-VHH constructs; we collected blood data for the IONP_PEG2000_ VHH triplet but not the IONP_PEG2000/750_ VHH triplet. Based on the findings that organ PK was essentially the same for these two IONP-VHH constructs, we used the blood data from IONP_PEG2000_ VHH triplet to constrain PK models for both constructs. This relies on the assumption that the blood clearance of the two IONPs were similar. Both models fit well, so this assumption seems reasonable.

There were several additional limitations. The precise stoichiometric relationships of iron oxide: PEG: VHH conjugates were not determined. We plan to use thermogravimetric analysis (TGA) to assess the nanoparticle stoichiometry in the future [69]. Also, opsonization/fouling of the nanoparticles in blood was not assessed in this study [70,71]. Our preliminary data (unpublished) indicate that similar PEG-coated IONPs remain stable in size over 24 h at 37 °C in human plasma, suggesting minimal fouling. Furthermore, we did not collect urine or feces, thus excretion was not directly measured. Finally, we did not systematically assess toxicity. Toxicity of IONPs is concentration and exposure time-dependent [72,73]. In general, iron oxide nanoparticles are considered very safe, but potential risks of iron can be related to oxidative stress and potential risks of foreign proteins can include immune responses [17,74]. Cationic iron can increase the production of reactive oxygen species (ROS), which may react and damage cell membrane and DNA. The cytotoxicity of our IONPs will be explicitly assessed in the future. We have not performed multidose experiments with long enough intervals between doses to assess for potential immune-related toxicity of the llama VHH proteins.

For future experiments, we will study the PK and distribution of the nanoparticles with adjusted components including VHHs targeting specific brain proteins and BBB components. PK and clearance of updated nanoparticle designs will be measured using the NIR imaging method and will be fitted using the PK compartment model developed in this paper. More complex, possibly nonlinear, models including information about binding kinetics and binding capacities will also be developed. Such experiments will incorporate genetically manipulated mice expressing human brain proteins and BBB components. Thus, for experiments involving complex genetically manipulated animals, methods that reduce the number of mice needed are especially relevant. Differences between genetically manipulated mice and appropriately matched controls will help reveal whether there are differences in in vivo kinetics and clearance, indicating target engagement of nanoparticles with biomarkers in brain. The mathematical PK models will be used to design experiments involving injecting the optimized nanoparticles into mice and imaging the signal change in brain using MRI at time points after injection selected based on the PK modeling. Similarly, the radiological-pathological correlations between in vivo imaging findings and ex vivo histology results will be studied at time points selected based on the model results.

## 4. Materials and Methods

### 4.1. Synthesis of VHHs

For this study, we synthesized VHH monomers of a nanobody called NB3 that did not bind any targets in wild-type mice using methods similar to those described in Esparza. et al. [30]. In addition, we produced a single polypeptide VHH heterotrimer that consisted of NB3, a (GGGS)_3_ linker; NB3, a (GGGS)_3_ linker; and another nanobody called H1 synthesized by Esparza et al. that also does not bind any targets in wild-type mice. The sequence and characteristics of these VHH constructs will be reported separately. These constructs were termed “VHH singlet” and “VHH triplet.” Phagemid pHEN2 with VHH triplet or VHH singlet were transferred into the BL21(DE3)-competent E. coli cells (C2527I, New England BioLabs, Ipswich, MA, USA) (Figure S10). The competent cells were grown in terrific broth medium at 37 °C. Isopropyl β-d-1-thiogalactopyranoside (IPTG) at a final 1 mM concentration was added to induce VHH expression when the OD_600_ reached 0.6. Following overnight expression, cells were pelleted by centrifugation and VHHs were extracted through periplasm extraction [30,75]. The 6x histidine-tagged VHHs were purified by Fast Protein Liquid Chromatography (FPLC) using a HisTrap™ FF Ni-NTA column (Cytiva, Marlborough, MA, USA). To further purify VHHs, they were size fractionated using a Superdex™ 75 10/300 GL column (Cytiva, Marlborough, MA, USA) with Phosphate Buffered Saline (PBS), pH 7.4, at a flow rate of 1 mL/min.

### 4.2. Conjugation of FNIR Dye to VHH Constructs

The NIR dye FNIR-Tag-NHS was provided by the Schnermann group [61]. Lysine groups on VHHs were labeled with FNIR dye through standard NHS conjugation. For conjugation of FNIR dye to VHH, a 1:1.5 molar ratio of VHH and FNIR dye were incubated together at room temperature for 2 h. Following conjugation, the VHH-dye conjugate was purified from unincorporated label using a 5 mL HiTrap^®^ desalting column (Cytiva, Marlborough, MA, USA) with PBS. Total protein concentration was measured using the Epoch microplate spectrophotometer (BioTek, Winooski, VT, USA) by measuring absorption at wavelength 280 nm and corrected using the theoretical extinction coefficient.

### 4.3. Production of IONPs, Ligand Exchange, and Conjugation of VHH Constructs

IONPs were generated by the thermal decomposition and ligand-exchanged methods described by Kim, et al. [9] (Supplementary methods). Two different approaches to ligand exchange were used, involving (1) PEG-Azide-2k, MW 2000 Da (Nanosoft Polymers, Winston-Salem, NC, USA) and (2) a 1:1 ratio of PEG-Azide-2k and PEG methyl ether, MW 750 Da (Sigma Aldrich, St. Louis, MO, USA). We developed two types of NPs. The first one was IONP_PEG2000_, which contained only PEG-Azide (MW 2000 Da) on the surface while the second type IONP_PEG2000/750_ comprised of both PEG-Azide (MW 2000 Da) and methoxy-PEG-ether (MW 750 Da). Solid state IONP_PEG2000_ and IONP_PEG2000/750_ were dissolved in 0.9% saline + 0.05% tween 80 solution and sonicated for 15 min followed by filtration through a 0.22 µm syringe filter. Then, a Superose™ 6 Increase 10/300 column (Cytiva, Marlborough, MA, USA) was used for size exclusion chromatography (SEC). Fractions corresponding to the eluted peak were collected. Hydrodynamic size of IONP was measured using the Dynapro^®^ Nanostar^®^ cuvette-based DLS instrument (Santa Barbara, CA, USA). FNIR dye and Dibenzocyclooctyne-amine (DBCO) were conjugated to VHH triplets at 1:1.5 molar ratio through standard NHS conjugation as described in Section 4.2. Peak fractions of IONPs were concentrated and conjugated with FNIR dye and DBCO-labelled VHH triplets via copper-free click chemistry for 24 h at room temperature [76,77].

The click chemistry allowed the reaction of DBCO-conjugated VHH triplets to the azide groups at the terminus of the PEG2000, yielding covalent attachment of VHH triplet-DBCO to PEG-Azide-2k ligand-coated IONPs (Figure S11d). After 24 h incubation, the click-reaction was resolved through a Superose 6 Increase column with 0.9% saline + 0.05% tween 80 solution for size exclusion separation of unreacted constituents. Fractions at the peak of size exclusion were collected and their size was measured using DLS. The peak fractions corresponding to VHH-conjugated IONP were used for in vivo pharmacokinetic studies (Figure S1).

### 4.4. Animals

All animal experiments were conducted under protocols approved by the National Institute of Neurological Disorders and Stroke (NINDS)/National Institute on Deafness and Other Communication Disorders (NIDCD) Animal Care and Use Committee in the National Institutes of Health (NIH) Clinical Center. C57BL/6J female mice were purchased from Jackson labs at 6–12-weeks of age and used at 7–12 weeks of age. Twenty mice were randomized into four groups with four to six mice in each group. Anesthetized mice were injected via tail vein with VHH singlet (five mice), VHH triplet (four mice), IONP_PEG2000_ VHH triplet (five mice), or IONP_PEG2000/750_ VHH triplet (six mice) for PK studies. Experiments were performed at the same time each day. The dark color fur coat of the strain of mouse used impedes the penetration of NIR light; therefore, the fur overlaying the regions of interest was removed by applying a topical depilatory cream above the brain, on the ventral and dorsal torso and tail of the mice 1 day prior to IV injection. Delayed skin hyperpigmentation was observed starting 4 days after hair removal.

### 4.5. Injection of Nanoparticles

Mice were anesthetized with 60% oxygen/40% medical air gas mixture containing 5% isoflurane in an induction box. After a stable anesthesia plane was established, mice were maintained at 1.5–2% isoflurane level. Artificial tears ointment was applied to prevent eye injury due to drying. Mice were placed on an electrical heating pad to maintain body temperature. Nanoparticle conjugates (in 0.9% saline + 0.05% tween 80 solution) and VHHs (in PBS) with different sizes (12 µM VHH singlet, 4.75 µM VHH triplet, IONP_PEG2000_ VHH triplet, IONP_PEG2000/750_ VHH triplet) were injected intravenously through single bolus injection at 0.1 mL volume into mice through the tail vein using 30 Gauge needles. Mice were maintained under anesthesia for approximately 3 min. Following the procedure, the mice were allowed to recover on a heating pad until fully ambulatory and then returned to their home cage with immediate access to food and water.

### 4.6. Multidose Experiment

VHH triplet was used for the multidose experiment. Five mice were injected, as described above, with 0.1 mL VHH triplet three times with 5 min intervals between each dose. Mice were maintained under anesthesia for approximately 3 min during intravenous injection and imaging and allowed to recover between injections.

### 4.7. NIR Imaging Methods

**Figure 9:**
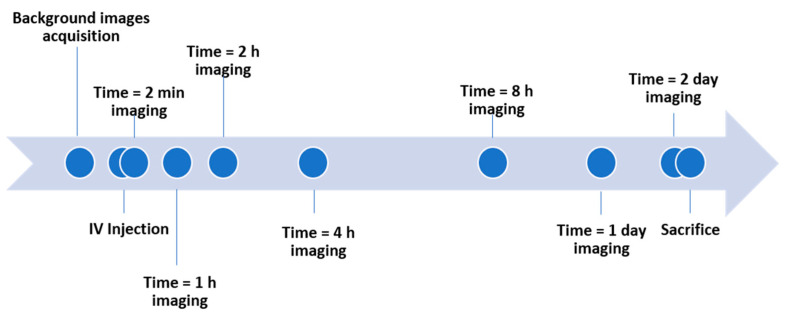
The experimental timeline of IV injection of VHHs and IONPs in mice. Background images were taken before injection, then NIR images were taken at 2 min, 1 h, 2 h, 4 h, 8 h, 24 h, and 48 h after injection. Mice were sacrificed after imaging at 48 h post injection.

Mice were anesthetized with 60% oxygen/40% medical air gas mixture with 5% isoflurane for induction of anesthesia and 1.5% isoflurane level for maintenance. Mice were imaged using the Pearl Trilogy Near-Infrared Fluorescent and Bioluminescent small animal imaging system (Licor, Lincoln, NE, USA). The specific parameters for NIR imaging were resolution = 170 um, fluorescence channel at 800 nm (excitation at 785 nm and emission at 820 nm) for FNIR dye. NIR images were collected before and at time 2 min, 1 h, 2 h, 4 h, 8 h, 1 day, and 2 days post IV injection (Figure 9) using Pearl. After end-point imaging, mice were euthanized by transcardial perfusion using 1X PBS with heparin under 5% isoflurane.

### 4.8. Image and Data Processing

Equally sized ROIs were manually drawn using the software Image Studio (version 5.2, Licor, Lincoln, NE, USA) from Li-Cor. Examples of ROIs are shown in Figure 1. Brain and right kidney ROIs were drawn from the prone view images while liver and front left paw ROIs were drawn from the supine view images. The average fluorescence intensity of each ROI was calculated and generated by Image Studio (version 5.2, Licor, Lincoln, NE, USA).

### 4.9. Blood Clearance Measurement

Twelve mice (six for each VHH) were injected with VHHs (VHH singlet, VHH triplet) and five mice were injected with nanoparticle IONP_PEG2000_ VHH triplet as described in the Section 4.5. After intravenous injection, mice were sacrificed at 1 min, 5 min, 10 min, 15 min, 30 min, and 1 h for mice injected with VHHs and at 1 min, 5 min, 15 min, 30 min, and 1 h for mice injected with IONP_PEG2000_ VHH triplet. An amount of 0.1–0.2 mL blood was collected from the right atrium into heparin-coated 1.5 mL microcentrifuge tubes. The microcentrifuge tubes with blood were imaged using the Pearl system and ROIs were drawn using the Image Studio software (Figure 4).

### 4.10. Mathematical Modeling

Mathematical models were built using MATLAB (The Mathworks, Inc., Natick, MA, USA) to predict distribution and pharmacokinetics based on nanoparticle size. The mathematical models were built based on kinetic data from VHH singlet, VHH triplet, IONP_PEG2000_ VHH triplet, and IONP_PEG2000/750_ VHH triplet data. Model fitting was done by minimization of the residual sum of squares across all five compartments and the blood clearance rate using the particle swarm algorithm [78,79]. r^2^ values were calculated to evaluate the goodness of fit. This model consists of five compartments: compartment 1 is the blood compartment, with a single bolus input and exchange of nanoparticles/VHHs with compartment 2 (kidney compartment), compartment 3 (liver compartment), and compartment 4 (brain 1 compartment). Compartment 4 also exchanges with compartment 5 (brain 2 compartment). In this model, the two main paths for clearance are the kidney and liver compartments. The kidneys and liver are the two main organs that are responsible for nanoparticle elimination [64]. The intake and clearance of nanoparticles from blood to kidneys/liver are the main characteristics that differentiate the in vivo kinetics of different nanoparticles and VHHs. The signal from spleen was not analyzed because of low signal strength. This five-compartment model was modified based on a four-compartment model with blood, liver, and kidney compartment as well but only one brain compartment. Based on AICc value [80] and model fitting, the five-compartment model was selected over the four-compartment model. Two compartments were used to better describe the nanoparticles/VHHs in vivo distribution and kinetics in the brain to guide the timing of future brain MRI studies.

## 5. Conclusions

This study investigated the PK of two VHHs and two VHH-conjugated iron oxide nanoparticles for their in vivo biodistribution and clearance in mice. A near-infrared method was established to monitor and record VHHs and VHH-IONPs kinetics in vivo. These results build a foundation for efficient understanding of VHHs and VHH-IONPs biodistribution and pharmacokinetics using near-infrared imaging.

## Figures and Tables

**Table 1:** Five-compartment model-fitted parameters for VHH singlet, VHH triplet, IONP_PEG2000_ VHH triplet, and IONP_PEG2000/750_ VHH triplet.

	k12	k21	k13	k31	k14	k41	k45	k54	k10
VHH singlet	0.02060	0.0018	0.0317	0.0502	0.0302	0.0847	0.3678	0.9212	0.3000
VHH triplet	0.0173	0.0007	0.0225	0.0006	0.0232	0.0625	0.0027	0.0010	0.2000
IONP_PEG2000_ VHH triplet	0.0156	0.0019	0.0466	0.0019	0.0063	0.0058	7.51E-05	0.9870	0.0300
IONP_PEG2000/750_ VHH triplet	0.0155	0.0032	0.0836	0.0024	0.0072	0.0063	0.0011	0.0050	0.0150

**Table 2:** Five-compartment model fitting r^2^ values of blood, kidney, liver, and brain ROIs. Kidney and liver uptake/clearance ratios are also calculated for each molecule.

	r^2^ Blood	r^2^ Kidney	r^2^ Liver	r^2^ Brain	Kidney Ratio	Liver Ratio
VHH singlet	0.9888	0.9718	0.9802	0.9942	11.2928	0.6317
VHH triplet	0.9803	0.9829	0.7086	0.9975	25.7424	40.8006
IONP_PEG2000_ VHH triplet	0.9949	0.9918	0.9592	0.1556	8.38232	24.1638
IONP_PEG2000/750_ VHH triplet	0.9749	0.9831	0.9150	0.2268	4.8673	35.4626

## Data Availability

The data presented in this study are available on request from the corresponding author. The data are not publicly available due to pending intellectual property disclosures.

## Supplementary Materials

The following are available online at https://www.mdpi.com/article/10.3390/ijms22168695/s1.
