# Supplementary material for: Pharmacokinetics of Single Domain Antibodies and Conjugated Nanoparticles Using a Hybrid near Infrared Method
Source: Int J Mol Sci. 2021 Aug 13;22(16):8695. doi: 10.3390/ijms22168695 (PMC8395466; doi:10.3390/ijms22168695)
Supplement: Supplementary file 1 [file ijms-22-08695-s001.zip › ijms-1299285-supplementary.pdf]

Pharmacokinetics of Single Domain Antibodies and Conjugated Nanoparticles Using a Hybrid Near Infrared Method

Supplementary Information

Authors: Shiran Su, Thomas J. Esparza, Duong Nguyen, Simone Mastrogiacomo, Joong H. Kim& David L. Brody

SUPPLEMENTARY METHODS 2

SUPPLEMENTARY FIGURES 3

Supplementary Fig. 1: FPLC fractions of VHHs and IONPs as well as DLS measurement of IONPs 3

Supplementary Fig. 2: Representative image of background autofluorescence in a mouse imaged using the 700nm and 800nm channel with the Pearl system 4

Supplementary Fig. 3: Effect of different NIR dyes on *in vivo* PK 5-6

Supplementary Fig. 4: Stability of FNIR dye NIR signal 7

Supplementary Fig. 5: The test-retest reliability of ROI drawing 8

Supplementary Fig. 6: Schematic graph and equations for the 4-compartment mathematical model 9

Supplementary Fig. 7: Fitting of the 4-compartment model solutions to the experimentally measured fluorescence data 10

Supplementary Fig. 8: Prediction of multidose regimen using 4-compartment model using fitted parameters from single dose experiment 11

Supplementary Fig. 9: Representative PK data of brain and front left paw after VHH triplet injection 12

Supplementary Fig. 10: Schematic representation of VHH singlet and VHH triplet in pHEN2 phagemid vector 13

Supplementary Fig. 11: Synthesis of $IONP_{PEG2000}$ VHH triplet 14

Supplementary Fig. 12: Characterization of IONPs 15

SUPPLEMENTARY TABLES 16

SUPPLEMENTARY REFERENCES 17

SUPPLEMENTARY METHODS

Iron oxide nanoparticle synthesis

Briefly, iron oxide cores were synthesized by decomposition of iron oleate in oleyl alcohol (Sigma Aldrich) and diphenyl ether (Sigma Aldrich) (**Figure S11a**). The iron oxide cores were only soluble in organic solvents. To make the iron oxide cores water soluble, PEG-derivatized phosphine oxide (PO-PEG) ligands were synthesized and exchanged with oleic acid on the iron oxide core surface [1]. PO-PEG ligands were synthesized by mixing the phosphoryl trichloride ($POCl_3$) with poly(ethylene glycol) methyl ether (mPEG) in anhydrous dichloromethane modified based on Na et al. [1]. Then, ligand exchange was performed to coat iron oxide cores with Poly(ethylene glycol) (PEG) resulting in IONPs soluble in aqueous solutions (**Figure S11c**). Iron oxide core and the ligands were combined and dissolved in toluene and chloroform (ACS grade) and the solution refluxed at 75C° for 4hr for the ligand exchange reaction. When the reaction was complete, the solution was washed with dichloromethane and hexane following centrifugation to remove unreacted substance. The pellet contained IONP were dried under vacuum. IONPs were characterized using Transmission Electron Microscopy (TEM) for core size measurement and infrared spectroscopy (IR) was performed to confirm the success of ligand exchange by the presence of an azide specific spectral peak (**Figure S12**).

SUPPLEMENTARY FIGURES

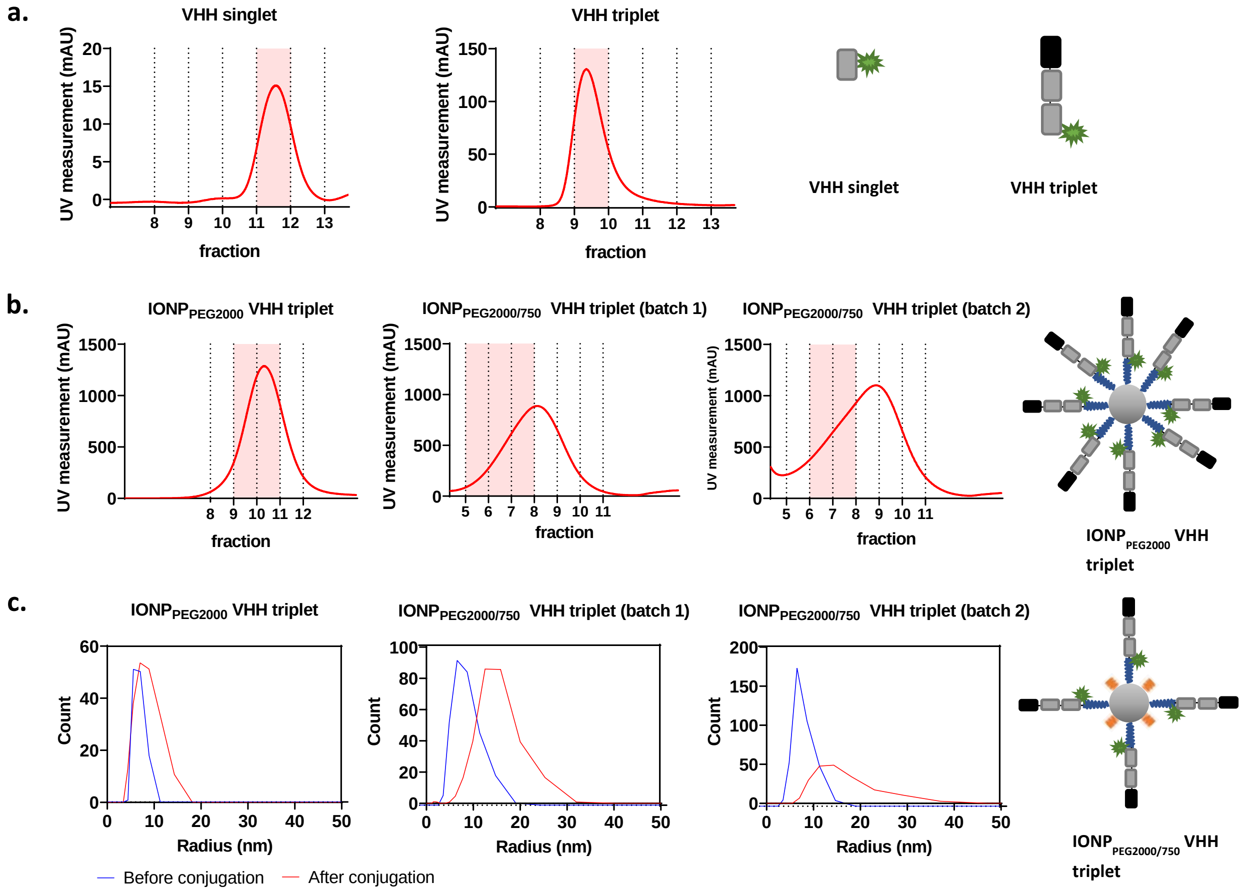

Figure S1. FPLC fractions of VHHs and IONPs as well as DLS measurement of IONPs. a. VHH singlet and VHH triplet fractions after passing through the Superdex 75GL size exclusion chromatography column. Elution fraction 11 for VHH singlet and fraction 9 for VHH triplet were collected for *in vivo* PK experiments (highlighted in pink). **b.** IONP$_{PEG2000}$ VHH triplet and IONP$_{PEG2000/750}$ VHH triplet fractions after passing through the Superose 6 Increase size exclusion chromatography column. Schematic drawing of IONP$_{PEG2000}$ VHH triplet with PEG2000 colored blue. Elution fraction 9 and 10 for IONP$_{PEG2000}$ VHH triplet, elution fraction 5, 6, and 7 for IONP$_{PEG2000/750}$ VHH triplet (batch1), 6 and 7 for IONP$_{PEG2000/750}$ VHH triplet (batch2) were collected for *in vivo* PK experiments (highlighted in pink). **c.** DLS hydrodynamic size measurements of IONP$_{PEG2000}$ and IONP$_{PEG2000/750}$ before (blue) and after (red) conjugation with VHH triplet. Schematic drawing of IONP$_{PEG2000/750}$ VHH triplet, with PEG2000 colored blue and PEG750 colored orange.

a. b.

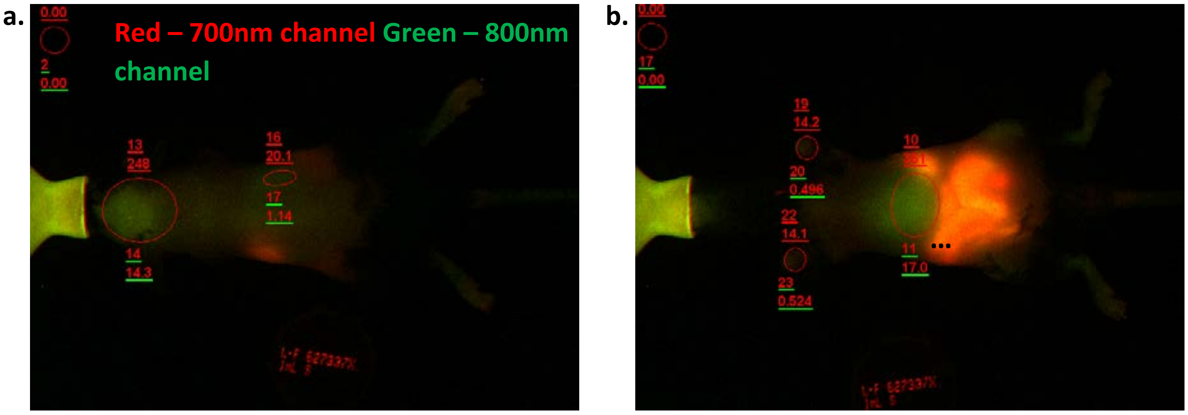

Figure S2. Representative image of background autofluorescence in a mouse imaged using the 700nm and 800nm channel with the Pearl system. a. Mouse background image in the prone position at 700nm (red) and 800nm (green) channel. **b.** Mouse background image in supine position at 700nm (red) and 800nm (green) channel. Autofluorescence at 700 nm is substantially higher than at 800 nm.

a. 700nm channel

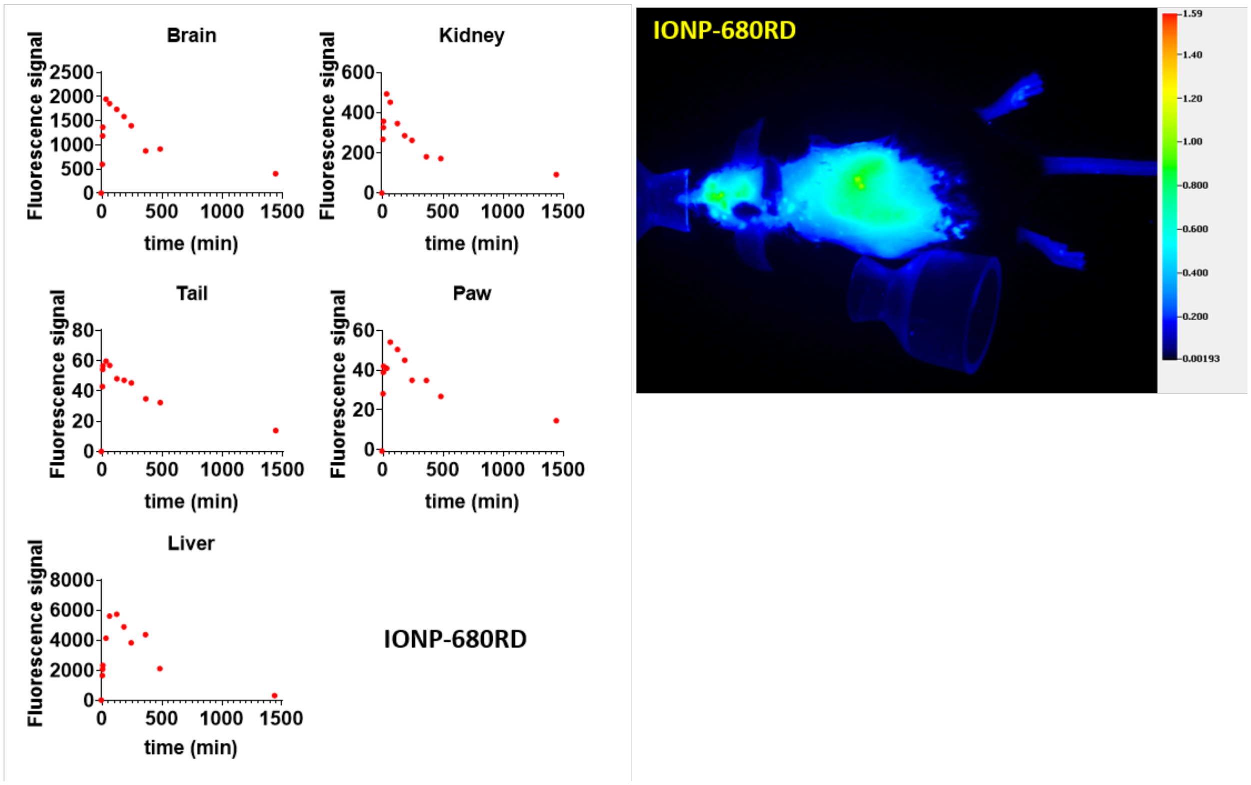

b. 800nm channel

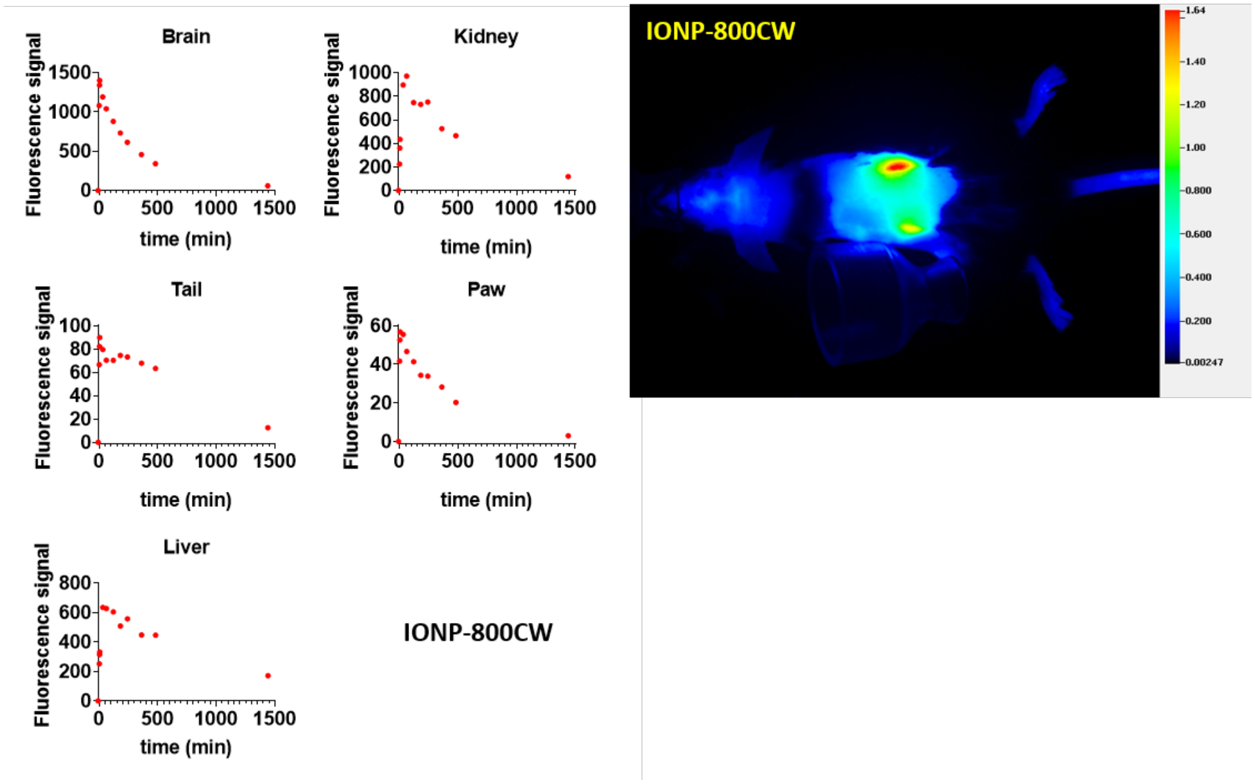

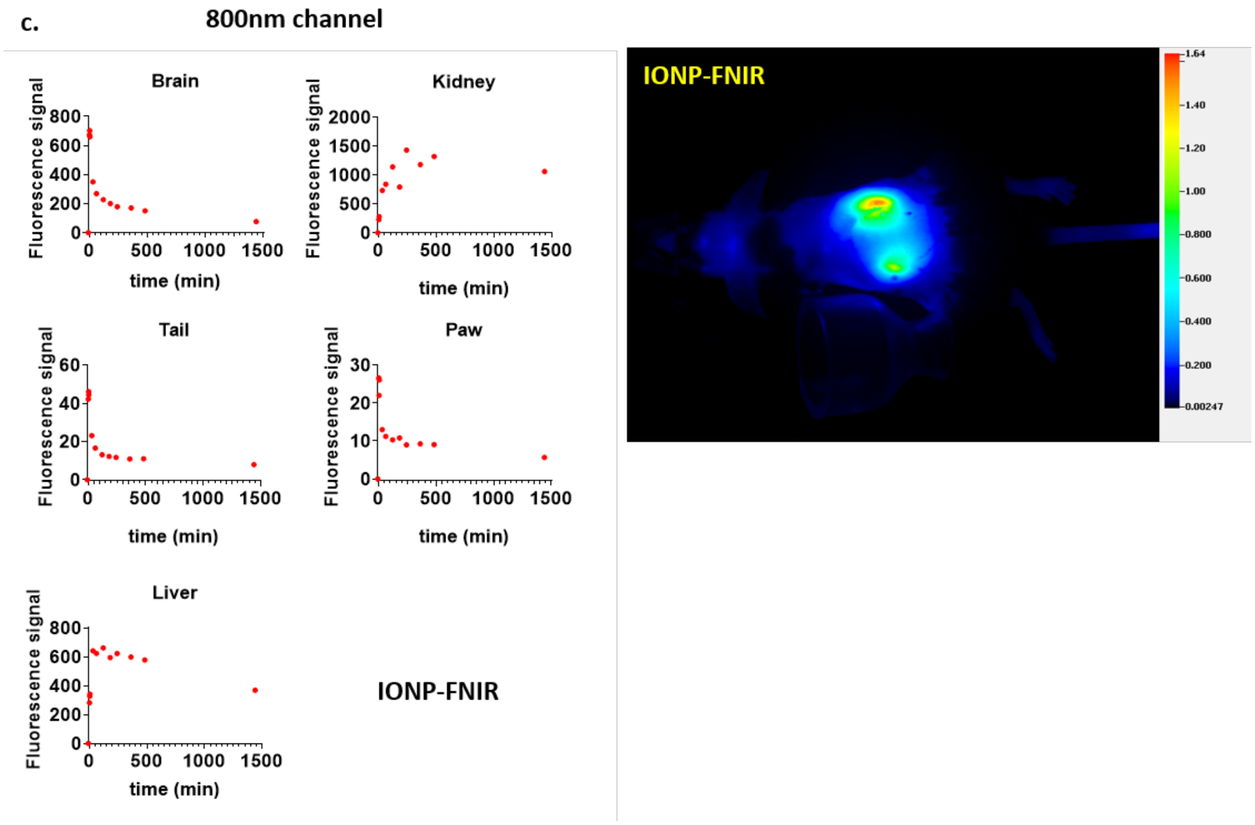

Figure S3. Effect of different NIR dyes on *in vivo* PK. Color bar represents NIR image intensity measured using the Pearl system. **a.** Representative data of mouse major ROI kinetics (Brain, Kidney, Tail, Paw and Liver) and representative NIR images of mice 1hr after injection of IONP-IR-680RD dye. **b.** Representative data of mouse major ROI kinetics (Brain, Kidney, Tail, Paw and Liver) and representative NIR images of mice 1hr after injection of IONP-IR-800CW dye. **c.** Representative data of mouse major ROI kinetics (Brain, Kidney, Tail, Paw and Liver) and representative NIR images of mice 1hr after injection of IONP-FNIR dye.

a. b.

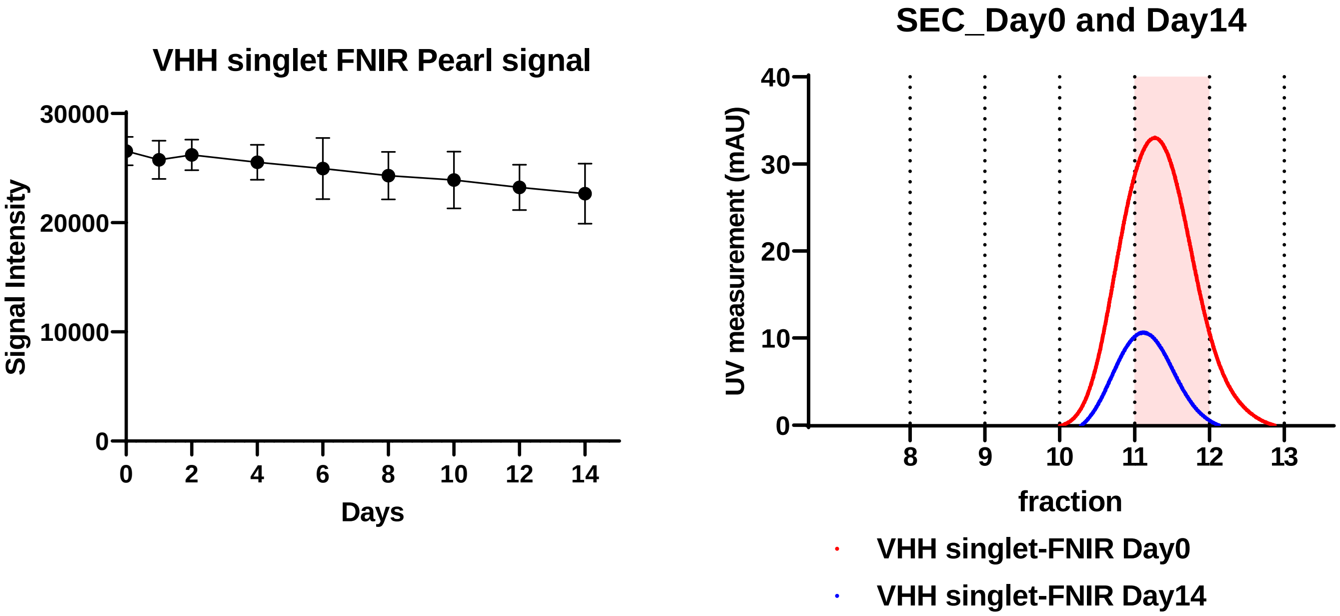

Figure S4. Stability of FNIR dye NIR signal. a. FNIR was conjugated to VHH singlet and the NIR signal was measured over 14 days using the Pearl system (n = 3). The FNIR dye signal was measured in the 800nm channel using the Pearl system. VHH singlet FNIR conjugate was kept in PBS at room temperature wrapped with aluminum foil to protect from light. The concentration of the VHH singlet FNIR conjugates were 6.85µM. Error bars represent standard deviations. **b.** FNIR was conjugated to VHH singlet and the size of VHH singlet-FNIR conjugate was measured with the Superdex 75 size exclusion column. VHH singlet-FNIR peaked at fraction 11 on both day 0 and day 14 after conjugation. The peak fractions on day 0 and day 14 were collected for NIR measurement as shown in **a.**

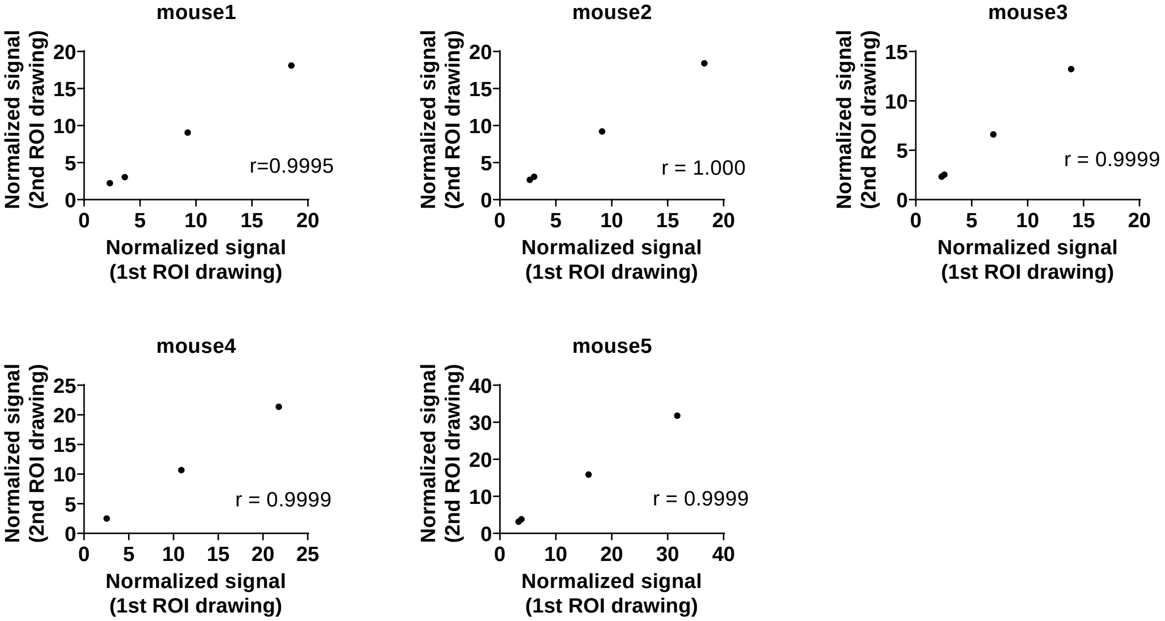

Figure S5. The test-retest reliability of ROI drawing. On a representative mouse injected with VHH singlet imaged 1hr after injection, major ROIs including brain, kidney, liver and left front paw were drawn two times to assess the test-retest reliability of ROI drawing. The Pearson correlations were calculated to be 0.9995, 1.000, 0.9999, 0.9999, 0.9999 for the 5 mice, indicating that the 1st and 2ndROI drawings give almost perfect reliability.

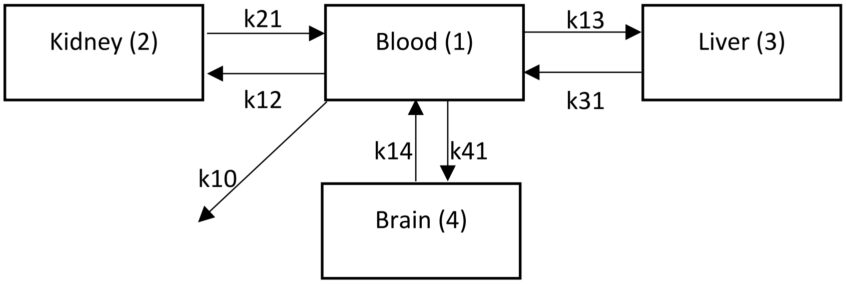

$$\frac{dX1}{dt} = -(k_{12} + k_{13} + k_{14})X_1 + k_{21}X_2 + k_{31}X_3 + k_{41}X_4$$

$$\frac{dX2}{dt} = -(k_{21})X_2 + k_{12}X_1$$

$$\frac{dX3}{dt} = -(k_{31})X_3 + k_{13}X_1$$

$$\frac{dX4}{dt} = -k_{41}X_4 + k_{14}X_1$$

Figure S6. Schematic graph and equations for the 4-compartment mathematical model. This schematic graph describes the *in vivo* kinetics of VHHs/IONPs, including uptake, clearance and intercompartment exchanges. This model is characterized by a system of four ordinary differential equations (ODEs). k12, k21, k13, k31, k14, k41 are the forward and reverse first-order transfer rate constants for the intercompartment change between blood compartment and kidney, liver, and brain compartments. k10 is the first-order rate constant for clearance.

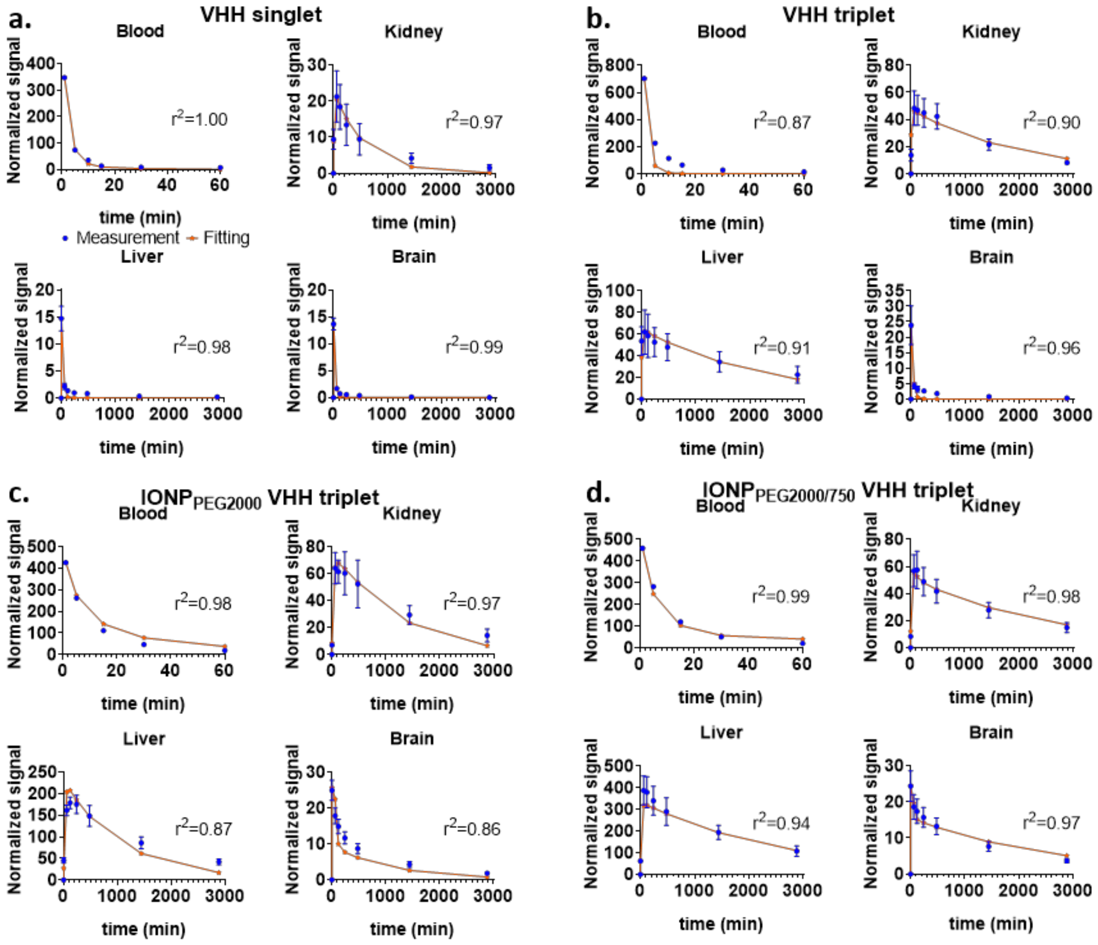

Figure S7. Fitting of the 4-compartment model solutions to the experimentally measured fluorescence data. Fitting of the model simulated solutions (red lines) and the measured normalized NIR signals (blue symbols) of major ROIs after IV injection of VHH singlet (**a.**), VHH triplet (**b.**), IONP$_{PEG2000}$ VHH triplet (**c.**) and IONP$_{PEG2000/750}$ VHH triplet (**d.**). The data is the same as that shown in Figure 6. Error bars represent standard deviations.

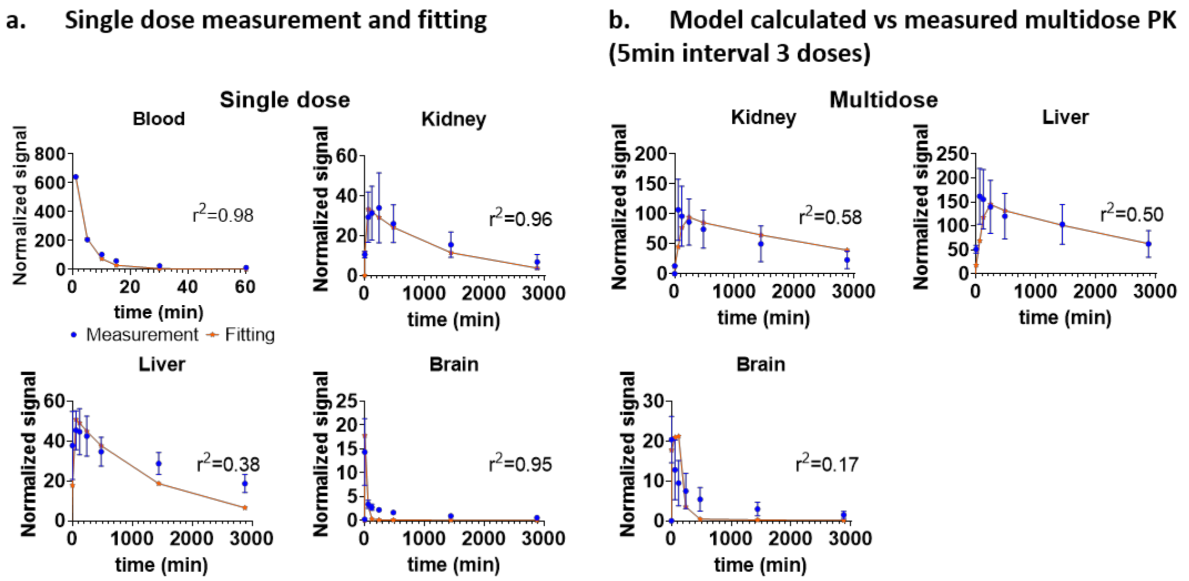

Figure S8. Prediction of multidose regimen using 4-compartment model using fitted parameters from single dose experiment. a. NIR signal change over time after single IV bolus injection (n=3 mice, blue dot) and the fitting solution (red line) calculated by the 4-compartment model. **b.** Experimentally measured fluorescence signal (n=5 mice, blue dot) and prediction of multidose signal (red line) based on the 4-compartment model single dose fit. Three bolus IV injections with time interval of 5 min between doses was performed for the multidose experiment. Error bars represent standard deviations.

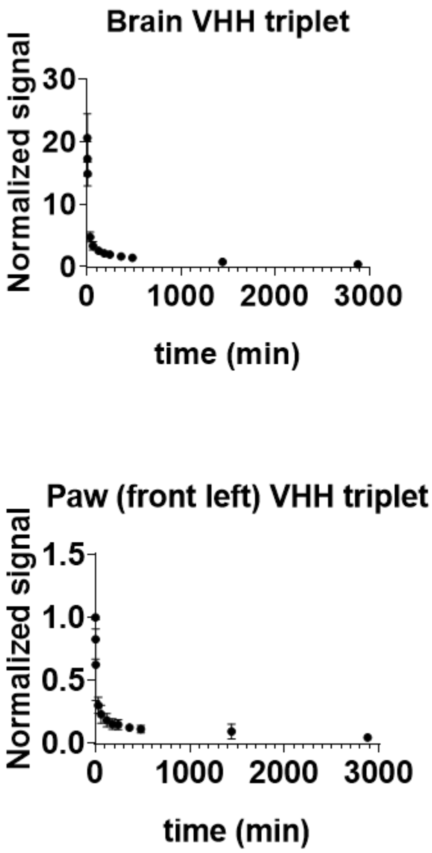

Figure S9. Representative PK data of brain and front left paw after VHH triplet injection. The brain and front left paw show similar PK (n = 6). Error bars represent standard deviations.

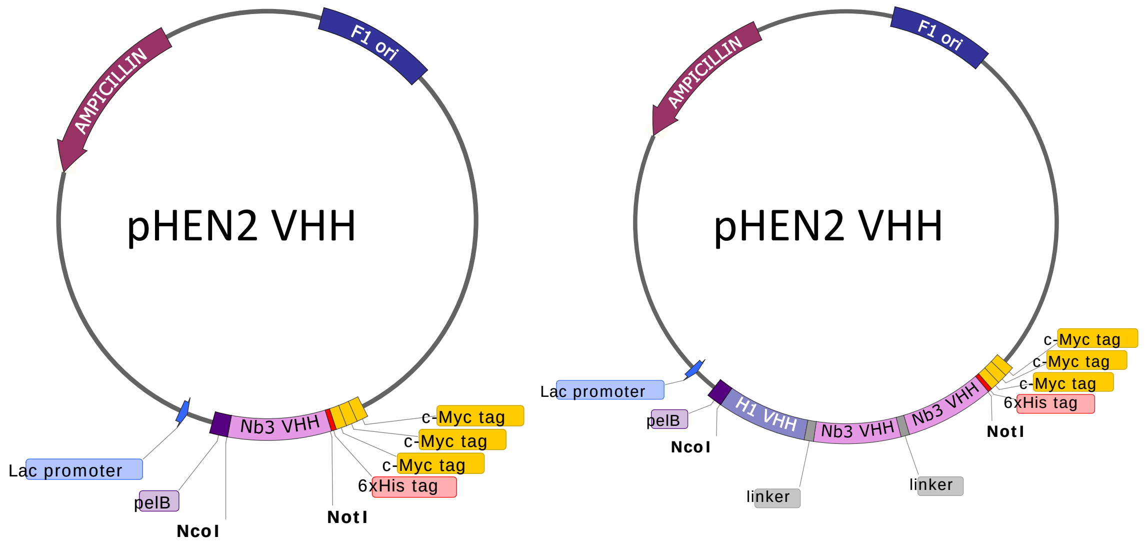

Figure S10. **Schematic representation of VHH singlet and VHH triplet in pHEN2 phagemid vector.** VHH singlet and VHH triplet DNA constructs were inserted into the pHEN2 phagemid vector for protein expression. For VHH triplet, the individual VHHs were linked using $(GGGS)_3$ linkers. This figure was generated using SnapGene software (from Insightful Science; available at snapgene.com).

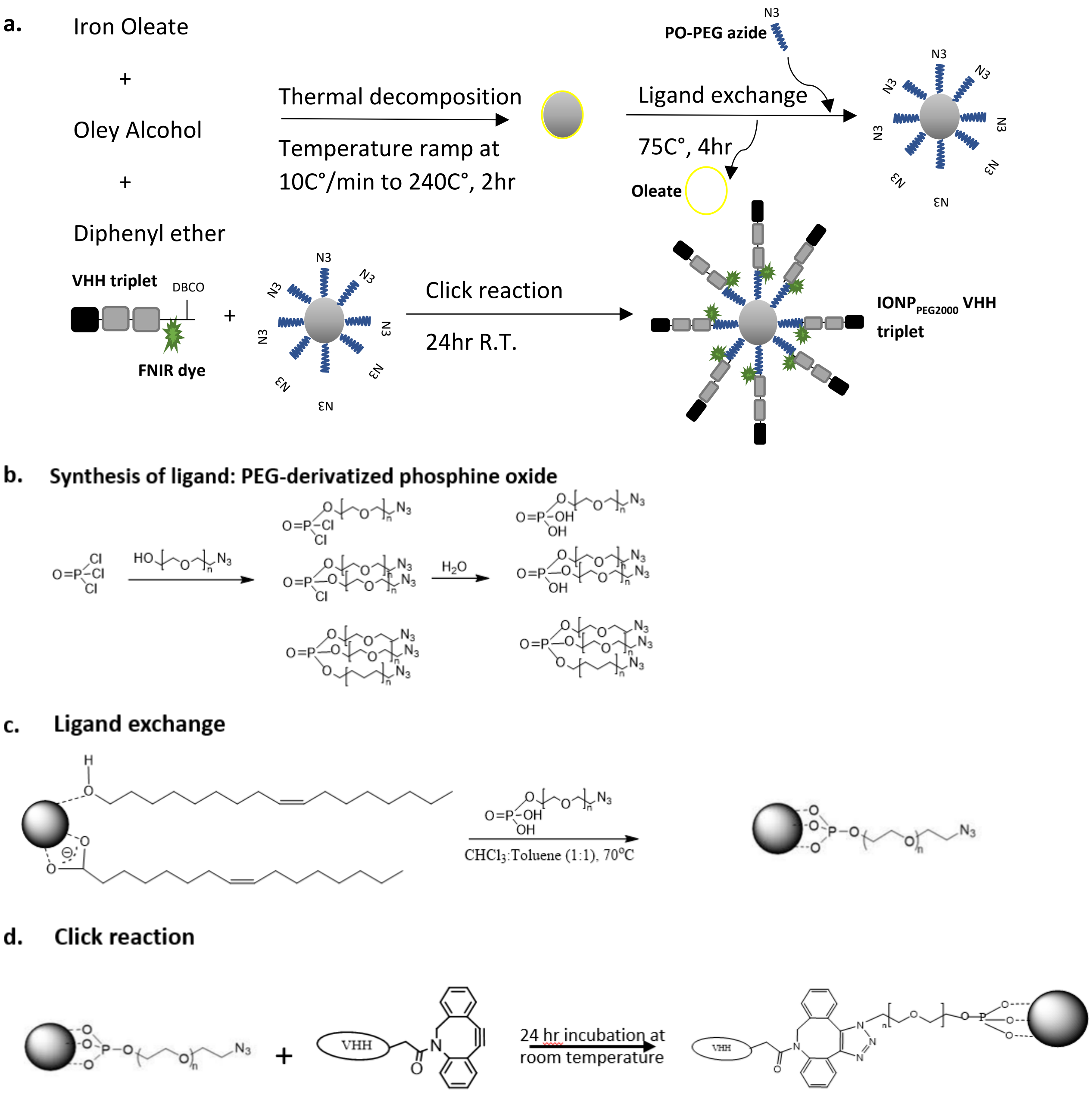

Figure S11. Synthesis of IONP$_{PEG2000}$ VHH triplet. **a.** IONP cores were produced through thermal decomposition, then ligand exchange was performed to coat IONP cores with PEG ligands. VHHs were labeled with FNIR dye and attached to ligand exchanged IONPs through click reaction with terminal azide (N_3). **b-d.** Chemical reactions describing PO-PEG ligand synthesis [1], ligand exchange reaction [2] which replaced oleic acid with PO-PEG ligands on the surface of the iron oxide core to make the IONP water soluble and click chemistry [3, 4] which conjugated VHH triplet to IONP. The ligand exchange and click reaction of di-substituted PO-PEG ligand and tri-substituted PO-PEG ligand are not shown here.

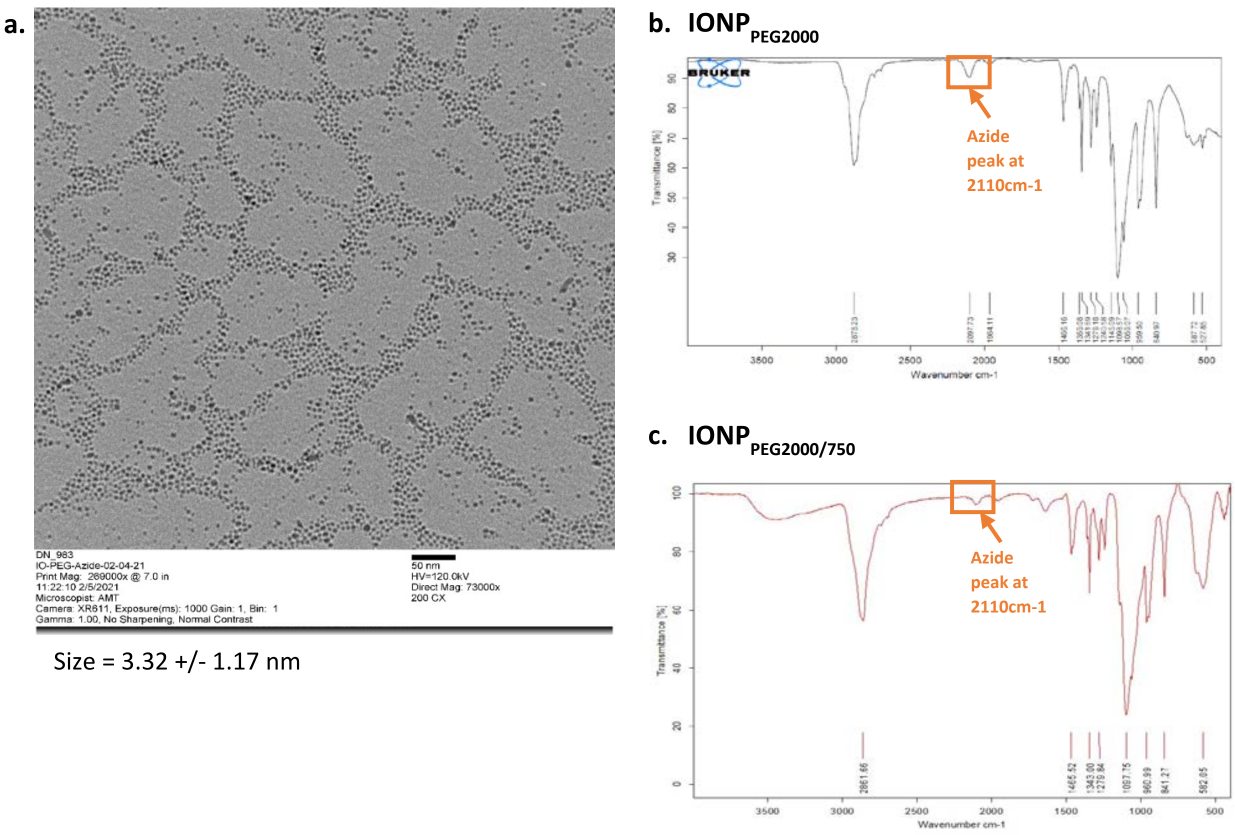

Figure S12. Characterization of IONPs. a. Transmission electron microscopy (TEM) image of a representative batch of iron oxide cores with diameter measured at 3.32 +/- 1.17nm. The iron oxide core size was quantified using ImageJ[5]. **b.** IR measurement of a representative batch of $IONP_{PEG2000}$. Azide peaks at 2110cm^{-1} before conjugation to VHH triplet. After conjugation, the azide is destroyed, as in Figure S11d. **c.** IR measurement of a representative batch of $IONP_{PEG2000/750}$. Azide peaks at 2110cm^{-1} before conjugation to VHH triplet. After conjugation, the azide is destroyed, as in Figure S11d.

SUPPLEMENTARY TABLES

Table S1. 4-compartment model fitted parameters for VHH singlet, VHH triplet, $IONP_{PEG2000}$ VHH triplet and $IONP_{PEG2000/750}$ VHH triplet.

	k12	k21	k13	k31	k14	k41	k10
VHH singlet	0.0172	0.0019	0.0304	0.0595	0.0284	0.0650	0.2446
VHH triplet	0.0324	0.0006	0.0439	0.0005	0.0280	0.0381	0.4007
$IONP_{PEG2000}$ VHH triplet	0.0110	0.0037	0.0356	0.0044	0.0360	0.0839	0.0141
$IONP_{PEG2000/750}$ VHH triplet	0.0153	0.0116	0.0800	0.0094	0.0329	0.0801	0.0046

Table S2. 4-compartment model fitting r^2 values of blood, kidney, liver, and brain ROIs. Kidney and liver uptake/clearance ratios are also calculated for each molecule.

	r^2 blood	r^2 kidney	r^2 liver	r2 brain	Kidney ratio	Liver ratio
VHH singlet	0.9968	0.9719	0.9809	0.9943	8.9935	0.5116
VHH triplet	0.8698	0.8985	0.9094	0.9584	54.0987	83.6981
$IONP_{PEG2000}$ VHH triplet	0.9790	0.9700	0.8734	0.8570	2.9838	8.1730
$IONP_{PEG2000/750}$ VHH triplet	0.9859	0.9843	0.9392	0.9744	1.3196	8.5452

SUPPLEMENTARY REFERENCES

1. Na, H.B., et al., *Versatile PEG-derivatized phosphine oxide ligands for water-dispersible metal oxide nanocrystals.* Chem Commun (Camb), 2007(48): p. 5167-9.
2. Kim, B.H., et al., *Large-scale synthesis of uniform and extremely small-sized iron oxide nanoparticles for high-resolution T1 magnetic resonance imaging contrast agents.* J Am Chem Soc, 2011. **133**(32): p. 12624-31.
3. Eeftens, J.M., et al., *Copper-free click chemistry for attachment of biomolecules in magnetic tweezers.* BMC Biophysics, 2015. **8**(1): p. 9.
4. Brudno, Y., et al., *In vivo targeting through click chemistry.* ChemMedChem, 2015. **10**(4): p. 617-20.
5. Schneider, C.A., W.S. Rasband, and K.W. Eliceiri, *NIH Image to ImageJ: 25 years of image analysis.* Nature Methods, 2012. **9**(7): p. 671-675.
